# Advances in Immunotherapeutic Approaches to Type 1 Diabetes

**DOI:** 10.3390/ijms24119220

**Published:** 2023-05-25

**Authors:** Annamaria Cudini, Alessandra Fierabracci

**Affiliations:** Bambino Gesù Children’s Hospital, IRCCS, 00165 Rome, Italy; annamaria.cudini@opbg.net

Type 1 diabetes mellitus (T1D) is a multifactorial autoimmune disease characterized by the selective destruction of pancreatic insulin-producing beta cells due to the aberrant activation of different immune effector cells (reviewed (rev.) in [1]). It is possible to identify three critical stages in the progression of T1D [2,3]. In the “first stage”, patients develop serum autoantibodies against beta cell antigens, which in turn activate CD4+ and CD8+ T cells, the main cells responsible for beta cell destruction. The second stage starts when insulin levels begin to decrease because of the destruction of beta cells by T cells. The third stage occurs in long-term patients, when there is a considerable reduction in C-peptide levels, which is an indicator of how much insulin is produced.

The latest Special Issue of the *International Journal of Molecular Sciences* entitled “Advances in Immunotherapeutic Approaches to Type 1 Diabetes” includes a total of seven contributions: 1 original article, 2 communications, and 4 reviews providing novel insights and information on immunotherapeutic strategies for insulin-dependent diabetes mellitus (type 1 diabetes, T1D).

The reviews by our group [4] and Cabello-Olmo et al. [5] discuss the most important immunotherapeutic interventions in T1D treatment in the pre-symptomatic and symptomatic stages of T1D established so far and those at advanced stages of evaluation. The immunological effects of different treatments either on T- or B-lymphocytes or on antagonizing the effect of proinflammatory cytokines are discussed.

The current T1D immunotherapies can be classified into four groups: antigen-independent strategies, antigen-dependent strategies, beta cell therapies, and stem cell therapies.

Antigen-independent strategies include antibody-based therapies, proinflammatory-cytokine-based therapies, and T regulatory cells (Tregs)-mediated therapies. Examples of the antibodies used for T1D treatment with some level of efficacy are abatacept, an anti-CTLA4 Ab (NCT01773707 trial ongoing), and the anti-CD3 monoclonal antibodies (MoAb) Teplizumab [6] and Otelixizumab [7]. Regarding inflammatory-cytokine-based therapies, the inhibition of these molecules is a successful strategy clinically used for the treatment of other autoimmune diseases, which may also be beneficial for T1D by inducing the preservation of pancreatic beta cells. Other non-antigen-specific interventions are Tregs-mediated therapies, whose aim is to expand this lymphocyte subset, thus increasing immune tolerance; autoreactive T- and B-cell-targeting therapies preserve pancreatic beta cell mass and restore insulin levels. In contrast, the advantage of autoantigen-targeting treatments could be the specific modulation of T1D-related autoimmunity without affecting the normal immune homeostasis. One possible strategy is beta cell autoantigen vaccination to achieve the induction of T cell tolerance against specific autoantigens. Since attempts to eliminate non-specific B cells, by treating T1D patients with anti-CD20 antibodies, have not been satisfactory, inhibition of specific autoantigen B cells could be a promising alternative [4].

The review by Cabello-Olmo et al. [5] further emphasizes the promise for the treatment of T1D presented by islet transplantation, which is part of the beta cell therapies group. Transplantation of pancreatic islets has several advantages, such as low morbidity and mortality, and it is more efficient in maintaining glycemic control without producing excess insulin [8]. On the other hand, adverse effects derived from the immunosuppression regimen, necessary for the transplantation process, represent an important issue to be considered. The satisfactory results derived from clinical trials have made it possible to test stem cell therapies. Newly diagnosed T1D patients who receive an infusion of autologous hematopoietic stem cells (HSCs) together with immunosuppression treatment show stable C-peptide levels and lower anti-GAD autoantibody levels. Furthermore, HSC transplantation seems to render patients insulin-independent from 6 months to 4 years after infusion [9,10]. At present, many trials are attempting to test the use of mesenchymal stem cells (MSCs) from different sources for the treatment of T1D since these stromal stem cells have important roles in tissue repair and regeneration [11].

Finally, in recent years, novel strategies have emerged for T1D immunotherapy. A potential treatment tool could be chimeric antigen receptor (CAR)–Tregs therapy, which consists of arming Tregs with beta-cell-specific CARs to increase Tregs’ migration into the pancreas, thus protecting the islet cells from autoimmune destruction [12]. The biggest challenge to the use of this exciting technology is the lack of beta-cell-specific antibodies that can be used to generate islet-protective CAR–Tregs. Novel publications discuss the role of the gut microbiota in diabetic pathology and report beneficial outcomes in mice models and humans following the administration of probiotics (rev. in [13]). In addition, recent evidence shows that IFN-driven JAK-STAT pathway activation significantly contributes to T1D pathogenesis, rendering the inhibition of the JAK-STAT pathway another potential immunotherapy strategy to be investigated [5].

Despite all of these possibilities, it is important to note that T1D is a very heterogeneous disease, and future therapeutic options will probably require a more personalized approach.

Lebenthal et al. [14] presented a phase II, double-blind, randomized, placebo-controlled, multicenter intervention trial in their study to assess the efficacy, safety, and tolerability of alpha-1 antitrypsin (ATT) as a therapeutic strategy for beta cell preservation in patients with recent-onset type 1 diabetes. Seventy T1D patients (between 8 to 25 years of age) were randomized in a 1:1:1 ratio to receive either intravenous AAT-60 mg/kg body weight, intravenous AAT-120 mg/kg body weight, or a matching placebo. Treatment with AAT was well tolerated as indicated by the fact that similar safety results were observed among the different groups. The primary outcome was the level of beta cell function at 52 weeks from baseline, as represented by the area under the curve (AUC) of the C-peptide from a 2 h mixed-meal tolerance test. The secondary outcome included glycemic control (as expressed by HbA1c levels), insulin dosage, hypoglycemic events, and safety parameters.

AAT treatment demonstrated a positive effect on beta cell preservation in the adolescent group (patients aged between 12 to 18 years of age) who received the higher dosage of AAT. Indeed, after 52 weeks, the C-peptide AUC levels in the adolescents receiving AAT-120 mg/kg body weight remained relatively stable in contrast to the decline observed in the placebo and AAT-60 mg/kg groups. Glycemic control, as expressed by mean HbA1c levels, was improved in the AAT-120 mg/kg-treated adolescents compared to the placebo and AAT-60 mg/kg groups.

However, these results should be interpreted with caution since the study population recruited was smaller than the calculated sample size, thereby reducing the statistical power of the trial by more than 20%.

Regarding the safety profile, the intensity of the adverse events that occurred was mild or moderate; furthermore, their severity and prevalence were independent of ATT dosage or the patient’s age. These data demonstrate the high safety profile of ATT treatment; however, its efficacy has yet to be determined.

The principal strengths of the study are the presence of a placebo group and the randomized design of the trial, while among the limitations, there are the absence of pharmacodynamic assays to determine the optimal dosage for achieving a therapeutic effect and a relatively small number of participants in each group.

Overall, this study suggests various options for beta cell recovery potential among patients from different age groups, thus underlining the need for individualized therapeutic intervention.

The review by Di Dedda et al. [15] discusses the effect of the pharmacological targeting of GLUT1 to control autoreactive T cell responses in T1D patients. The T cell response highly depends on bio-energetic metabolism, with important implications for translation into immunotherapies. In resting T cells, energy production is derived from oxidative phosphorylation, whereas activated T cells predominantly use the anabolic pathway of glycolysis to sustain cell growth and proliferation. Therefore, it is possible to exploit the metabolic differences of resting and activated T cells to target selectively autoreactive T cells in T1D.

One possible metabolic target is Glut1, the main glucose transporter for glucose uptake to fuel the glycolytic pathway in activated T cells. In particular, Glut1 is expressed at low levels on the surface of resting T cells and is upregulated upon T cell activation, causing increased glucose uptake which peaks at 48–72 h after activation [16]. The lack of Glut1 causes impaired proliferation of activated effector T cells which leads to defective generation of Th1, Th2, and Th17 cells both in vitro and in vivo, while it does not affect resting T cells and CD4+ CD25+ Tregs, indicating that Glut1 blockade could be an effective strategy to control T cell responses.

At present, a growing number of small molecules that act as selective inhibitors of Glut1 are becoming available, rendering pharmacological Glut1 blockade easily applicable to control autoreactive T cells in T1D. In particular, three small molecules have gained interest in recent years. STF-31, which binds directly to the Glut1 transporter, blocks glucose uptake and suppresses glycolysis in human T cells overexpressing Glut1 [17]. WZB117, which binds reversibly at the exofacial sugar-binding site of Glut1, inhibits glucose transport in T cells and reduces their proliferation by 90% (rev. in [15]). However, the clinical use of both STF-31 and WZB117 has significant limitations: they are indeed effective in the micromolar range and they have a controversial selectivity for Glut1. The most promising molecule for application in clinical settings is BAY-876, which is highly selective for Glut1, and it is characterized by an IC50 of 2 nM. Nevertheless, currently there are no data available regarding its effect on T cells (rev. in [15]).

As the majority of cells in the body express Glut1, it is important to evaluate potential off-target effects and side effects. In particular, there is an important diabetes-specific side effect to consider in a hypothetical T1D therapy based on Glut1 blockade, which is the potential inhibition of insulin production by beta cells; indeed, human beta cells express Glut1 and use it as a glucose sensor for optimal insulin secretion. Moreover, the authors concluded that the treatment duration and the patient’s age are critical aspects that need to be addressed to reduce toxicity and increase the therapy’s efficacy. In this scenario, treatment with Glut1 inhibitors could be introduced during the first two or three weeks following the transplantation of the islets or the pancreas to induce exhaustion of autoreactive and possibly alloreactive T cells.

Ludvigsson [18] reviewed recent updates on autoantigen treatment in T1D, highlighting unsolved questions on how to select autoantigens and the route of administration.

Autoantigen treatment is an “inverse vaccination”, with the aim of reducing or stopping a destructive specific autoimmune response by administering autoantigen(s) against which the autoimmune process develops. This type of treatment is conceptually the same as that used for allergic diseases: inducing tolerance against the allergens by exposing the immune system to a suitable dose of these molecules.

The advantages of autoantigen treatment are reduced toxicity and greater specificity than other forms of immune suppression/modulation; on the other hand, despite the evidence of antigen-specific immunotherapy’s efficacy in animal models, there is an urgent need to assess its efficacy in human trials. Moreover, several studies on autoantigen treatment for different autoimmune diseases, such as T1D, myasthenia gravis, rheumatoid arthritis, systemic lupus erythematosus, and celiac disease, have highlighted important issues that still need to be solved, i.e., selecting the appropriate autoantigen, identifying the optimal timing, dosage, and route for its administration, and finding biomarkers for monitoring efficacy.

T1D is a candidate autoimmune disease for autoantigen therapy, since autoantibodies and antigen-specific T cells can be detected in the peripheral blood of patients and high-risk individuals, thus rendering this treatment particularly interesting both to prevent T1D and to preserve residual beta cell function in patients with recent disease onset [19]. However, as regards the choice of autoantigen, several beta-cell-related autoantigens in T1D can be putatively considered; they can be divided into commonly discussed “old” autoantigens and recently described antigens/neoepitopes. The “old” autoantigens are insulin, glutamic acid decarboxylase (GAD65), insulinoma-associated protein 2/tyrosine phosphatase (IA-2), zinc transporter 8 (ZnT8), and proinsulin (rev. in [1]); the new ones are hybrid insulin peptides (consisting of fragments derived from both insulin and other insulin-secretory granule proteins) and neoepitopes (resulting from modifications during antigen processing and presentation) (rev. in [18]). Therefore, it may be useful to establish some criteria to select the most relevant antigens for autoantigen treatment, such as how a specific antigen fits the HLA type or to what extent the immune system evidently reacts against it. Another issue to consider is the route of autoantigen administration. Oral administration has the problem of protein degradation during the autoantigens’ passage through the gastro-intestinal tract (rev. in [18]); a possible solution, as shown at least in animal studies, is to deliver autoantigens encapsulated in bacteria, plant cells, or liposomes. Another route of administration is the intranasal route, which might make it easier for the antigen to be presented by the dendritic cells. Finally, considering the fact that antigens are presented by antigen-presenting cells (APCs) to immune T cells in the lymphatic tissue, a more effective route of autoantigen administration may be direct intra-lymphatic injection, but this method has not been described either in animal experiments or in human autoimmune diseases.

Several preclinical and clinical studies on insulin administration to prevent T1D have been performed and, whereas in the experimental animals intranasal insulin may prevent autoimmune diabetes, in human clinical trials, both intranasal and oral insulin show no efficacy. The subcutaneous injection of GAD bound to alum hydroxide (GAD–alum) was envisaged as a possible strategy to preserve beta cell function in newly diagnosed T1D patients. However, its therapeutic effect still needs to be improved, for example, by replacing subcutaneous administration with intra-lymphatic administration and by combining this strategy with other therapies.

The work by Orban et al. [20] proposes a novel quantitative approach for staging and evaluating recovery from T1D: the T1D metabolic recovery index. After 100 years since the discovery of insulin by Banting and Best (1921), T1D patients still develop short- and long-term complications. Even today, blocking the autoimmune process that causes pancreatic beta cell destruction remains a major challenge, despite the numerous immunotherapeutic approaches that have been proposed over the last 50 years. Alongside the need for effective treatment, it is important to have a tool that quantifies and predicts the stages of recovery from T1D, as well as a tool that can reliably compare therapies to find those with the most substantial beneficial impacts. For this purpose, Orban et al. [20] developed an equation to calculate the T1D metabolic recovery index (DMMRI) using outcome data from three recently published clinical trials. They combined three clinical parameters (C-peptide, HbAc1, and insulin dose) into one formula based on the concept that higher C peptide levels are linked to lower HbA1c levels and lower insulin usage. The value obtained by calculating this equation is a measure of the efficacy of the interventions: a value of 5 is indicative of no effect for the intervention; values over 5 mean proportional improvement; and values less than 5 signify proportional worsening of the metabolic status as a result of the intervention. Another potential utility of this index is to help in selecting the best combination therapies by choosing those with the highest indices. Currently, DMMRI has been tested in three trials: in the two successful trials, Rituximab and Abatacept obtained indices over 5, and the GAD vaccination trial resulted in ineffective outcomes and this conclusion is supported by a DMMRI of less than five. In the future, further testing will be needed to assess the utility of this index.

Recently, it has been demonstrated that beta cells actively participate in the pathological process of T1D by acquiring a senescence-associated secretory phenotype (SASP) (rev. in [21]). Generally, the SASP occurs in the context of persistent DNA damage response (DDR) signaling and is mediated by SASP factors. The genes that encode SASP factors are activated through extensive chromatin remodeling and modification. In particular, the SASP in pancreatic islet cells is transcriptionally regulated by epigenetic readers, bromodomain extraterminal (BET) proteins, including bromodomain-containing protein 4 (BRD4), both in humans and in mouse models. As highlighted by Thompson et al. [21] in their study, inhibition of BRD4 chromatin binding with the small molecule BET inhibitor iBET-762 leads to diminished SASP gene expression, protein secretion, and SASP paracrine activity from NOD mouse islets. Furthermore, treatment of NOD mice with the same inhibitor prevents diabetes and attenuates the SASP in the islets in vivo. However, it is important to consider that BET proteins are also expressed in some immune cell types; therefore, BET inhibitors could block immune inflammatory pathways unrelated to the SASP. An alternative strategy could be identifying islet-cell-specific transcription factors that interact with BRD4 in SASP genes regulation in order to target them and selectively inhibit SASP gene expression without affecting inflammatory responses. Further studies are necessary to better understand the mechanism by which BET proteins are recruited to the SASP gene regulatory regions in the islet cells. However, targeted inhibition of the SASP transcriptional program may represent a new opportunity for T1D management.
